# Successful in vitro propagation of feline coronavirus from clinically diagnosed feline infectious peritonitis cases using Vero cells: A potential model for future research

**DOI:** 10.1002/vro2.70030

**Published:** 2026-02-25

**Authors:** Eaftekhar Ahmed Rana, Mahfuza Akther, Susan Beetson, Subir Sarker, Gabriele Rossi, Jasim M. Uddin

**Affiliations:** ^1^ School of Veterinary Medicine Murdoch University Perth, West Australia Australia; ^2^ Department of Microbiology and Veterinary Public Health Chattogram Veterinary and Animal Sciences University Chattogram Bangladesh; ^3^ Biomedical Sciences & Molecular Biology, College of Medicine and Dentistry James Cook University Townsville Queensland Australia; ^4^ Centre for Animal Production and Health Food Futures Institute Murdoch University Murdoch, West Australia Australia; ^5^ Centre for Biosecurity and One Health Harry Butler Institute Murdoch University Perth, West Australia Australia

**Keywords:** cats, cytopathic effect, FCoV, laboratory diagnosis, phylogenetic analysis, RT‐qPCR, Vero cell culture

## Abstract

**Background:**

Feline coronavirus (FCoV) causes inapparent to progressive fatal feline infectious peritonitis (FIP) in domestic and wild cats, which affects multiple‐organ systems.

**Methods:**

We investigated three clinically sick cats using different laboratory and molecular tests to diagnose and confirm FCoV and propagate the virus in Vero cell culture.

**Results:**

All the cats exhibited effusive FIP with multiple clinical signs. The haematology profiles revealed lymphopenia in all cases and leukopenia, neutropenia and regenerative left shift in one case. Serum biochemistry showed elevated creatine kinase, aspartate aminotransferase, hyperbilirubinaemia and hypoalbuminaemia in all the cases. Urinalysis revealed bilirubinuria in two cases and marked proteinuria in another. All effused samples showed a positive Rivalta test, and the cytology showed a mixed pyogranulomatous inflammatory exudate. Reverse transcription quantitative polymerase chain reaction (RT‐qPCR) confirmed that all the cats were infected with FCoV. The specific gene sequencing of three isolates clustered in the same clade of the phylogenetic tree, suggesting a closely related genotype associated with FIP in cats. Feline coronavirus induced cytopathic effects (CPEs) in Vero cells, first appearing on day 5 post‐infection (pi) in the primary passage. In the second passage, more distinct CPEs, including cell rounding, shrinkage, detachment and death, were evident from day 2 pi. Reverse transcription quantitative polymerase chain reaction confirmed active viral replication, with significantly decreasing Ct values across passages.

**Conclusions and clinical importance:**

The adaptation and propagation of FCoV in a non‐feline cell line provide promising opportunities for future studies, including generating sufficient viral RNA for sequencing, evaluating antiviral resistance, and establishing a practical in vitro system for drug screening and vaccine development.

## INTRODUCTION

Feline infectious peritonitis (FIP) is a viral disease of domestic and wild cats, caused by infection with feline coronavirus (FCoV), which is fatal when untreated. Two forms of the aetiological agent FCoV, commonly referred to as biotypes, are recognised: feline enteric coronavirus (FECV) and feline infectious peritonitis virus (FIPV).^1^ A striking feature of FIPVs, distinguishing them from FECVs, is their pronounced ability to efficiently replicate in circulating monocytes and macrophages. Feline infectious peritonitis virus is extremely pathogenic and arises from mutations in the avirulent, highly contagious FECV, which facilitates systemic spread with severe immunoinflammatory consequences in the host body.^2^ The FIPV induces severe vascular inflammation, which may result in a wide variety of clinical signs and rapidly develop into a serious and fatal condition.^1^ The disease remains a leading cause of mortality in young cats globally, including in Australia.^3^ Thus, an early diagnosis is crucial to promptly initiate the clinical therapy and management.

Feline coronavirus is a single‐stranded positive‐sense (+) RNA virus belonging to the family *Coronaviridae* under the genus *Alphacoronavirus*. Feline coronavirus has two distinct genotypes,^4^ FCoV‐1 (previously serotype I) and FCoV‐2 (previously serotype II), classified based on antigenic and genetic differences in the spike (S) protein.^1^, ^4^, ^5^ Due to its genetic structure and high mutation rate, FCoV exhibits significant genetic diversity, which influences its global distribution and pathogenic potential.^6^ Although virus propagation in susceptible cell lines is primarily the gold standard for virological studies and diagnosis, it is also essential for exploring virus‒host interactions, characterising immune responses, assessing both existing and novel therapeutic agents, and designing effective vaccines. Notably, FCoV‐1 is generally difficult to propagate in vitro due to its poor tropism for different cell lines, and the cellular receptor for viral attachment remains unclear.^6^ Therefore, it often requires feline macrophage‐derived cultures for propagation^5^; even in these systems, replication is typically poor, posing challenges for laboratory studies, including effective antiviral and vaccine development efforts. In contrast, FCoV‐2 exhibits a greater capacity for in vitro adaptation, replicating efficiently in feline‐specific cell lines such as Crandell‐Rees Feline Kidney (CRFK) and feline whole foetus (FCWF) cells, and demonstrating the ability to adapt to and replicate in certain non‐feline cell lines as well.^5^, ^7^ Among them, Vero cells are a continuous cell line derived from African green monkey kidney epithelial cells. Vero cells express viral entry receptors, are highly permissive to diverse mammalian viruses, and are widely used in vitro for virology studies due to their availability and ease of culture. However, the suitability of FCoV propagation in non‐feline mammalian cell lines, such as widely available and easily cultured Vero cells, is unknown.

Furthermore, the diverse clinicopathological manifestations of FIP pose significant diagnostic challenges for clinicians, especially when using non‐invasive approaches. A definitive diagnosis of FIP requires consistent clinical signs, supportive histology or cytology, and detection of the virus within macrophages via immunostaining, such as immunohistochemistry or immunocytochemistry, which has been reported to provide high specificity for confirming FCoV.^3^, ^8^ Sometimes, a single test may not reliably provide a conclusive diagnosis of FIP; thus, the antemortem diagnosis remains challenging to confirm the disease.^1^ These challenges focus on the need for a combination of clinical examination, haematological, biochemical and cytological evaluation, and advanced diagnostic techniques to improve diagnostic accuracy.^8^ Moreover, confirmatory antemortem diagnosis of FIP depends on the detection of FCoV‐specific antibodies or antigens, as well as the identification of genetic markers and the successful propagation of the virus in susceptible cell culture systems.^9^, ^10^, ^11^ Moreover, the commercialised reverse transcription quantitative polymerase chain reaction (RT‐qPCR) and sequencing approaches using effusion samples provide veterinarians with a non‐invasive and reliable tool for definitive FIP diagnosis, where a positive result is highly specific and indicative of the disease. Thus, without a confirmatory diagnosis, the clinical management of FIP in cats is often difficult, frequently resulting in poor prognosis and fatal outcomes.^8^, ^9^ However, once FIP is confirmed, specific antiviral and supportive therapies can significantly improve survival in affected cats.^10^, ^11^ Notably, recent advances in antiviral therapy, particularly with nucleoside analogues such as GS‐441524, remdesivir and molnupiravir, have shown significant therapeutic promise.^10,11^ For this reason, this study investigated three clinically sick cats, each with distinct findings, to evaluate their clinicopathological features and perform detailed laboratory testing and molecular analyses, primarily to confirm FCoV infection and select cases for in vitro cell culture studies. To date, there are limited reports of in vitro propagation of FCoV using widely used continuous cell line, such as Vero cell culture, leaving a critical gap in virus characterisation that contributes to advancements in virology research. Therefore, the primary objective of this study was to evaluate and characterise the in vitro propagation of wild‐type FCoV (naturally occurring strains circulating in cats) using Vero cells, providing a promising cell culture system for virus isolation and enhancing our understanding of FCoV dynamics.

## MATERIALS AND METHODS

### Clinical cases and sampling

Three clinically sick cats were admitted to The Animal Hospital at Murdoch University (TAH‐MU) for clinical diagnosis and treatment purposes. A careful clinical examination of the cats was performed based on their clinical history and presenting signs (Table S1). For laboratory diagnosis, 3‒5 mL of peritoneal effusion and 3 mL of peripheral blood were collected into an EDTA and a plain tube. The samples were immediately submitted to the VetPath Laboratory Services, TAH‐MU. Notably, the first clinical case (designated as cat 1) was a 12‐month‐old male mixed medium hair cat, the second case (designated as cat 2) was a 6‐month‐old male British shorthair cat, and the third case (designated as cat 3) was a 12‐month‐old male Domestic shorthair cat. All three clinically affected cats were identified between July and September 2024 and originated from the same geographical region within the city of Perth, Western Australia.

### Haematology, serum biochemistry and serological analyses

Haematological test was performed using an automated laser cell analyser (Sysmex XN‐1000 V) and serum biochemical parameters were estimated using an automated spectrophotometer (DxC 700 AU, Beckman Coulter). The haematology and biochemistry parameters measured are listed in Table S2. The presence of FCoV‐specific antibody titre in blood serum was tested using indirect immunofluorescent antibody tests (IFATs) described by Addie et al.^12^ The assay was performed using the FCoV IFAT Kit (Thermo Scientific) following the manufacturer's instructions. Serum samples with titres of 1:40 or more were considered seropositive, while those with titres of 1:10 or less were considered seronegative. Slides were examined under a fluorescence microscope, and results were interpreted according to the manufacturer's guidelines. Moreover, *Toxoplasma gondii* IgM antibodies were assessed using an IFAT to identify recent or acute infections, with titres of 1:64 or more considered positive, to aid in the differential diagnosis of overlapping infections.

### Analysis of urine and peritoneal effusion samples

Urine samples were collected via cystocentesis, and specific gravity as well as the biochemical analysis were performed following routine protocols using a refractometer and a reagent strip (Multistix 10 SG, Siemens), respectively, as listed in Table S3. Urine sediment was examined at 400× magnification to identify and enumerate red blood cells (RBCs), white blood cells, epithelial cells, bacteria, yeast/fungus, parasites and small crystals.

For peritoneal effusion fluid, total nucleated cell count and RBC count were measured using a manual haemocytometer (Neubauer counting chamber), mentioned in Table S4. Subsequently, protein concentration was measured using the pyrogallol red colorimetric method on an automated spectrophotometer (DxC 700 AU, Beckam Coulter). Rivalta's test was performed to differentiate between exudative (inflammatory) and transudative (non‐inflammatory) effusion fluids using the protocol described by Tasker.^9^ For cytological evaluation, both direct smear and 50 µL cytospin preparations were examined. All slides were stained with modified Wright's stain using a semiautomated slide stainer for laboratories (Hematek 3000, Siemens).

### Bacterial and fungal culture analysis

Freshly collected effusion fluid samples were subjected to bacterial and fungal culture tests using 5% sheep blood agar and Sabouraud dextrose agar (SDA). In total, 20 µL samples were inoculated on both agar plates and incubated at 37°C under aerobic conditions to evaluate bacterial and yeast growth. Plates were examined at 24‐h intervals for up to 72 h to assess bacterial growth and up to 7 days to observe yeast growth. Additionally, 200 µL of each sample was inoculated onto SDA plates and incubated at 25°C aerobically for 7 days to observe the mould's growth.

### RT‐qPCR for FCoV detection

In total, 300 µL peritoneal effusion samples underwent viral RNA extraction using a commercial RNA isolation kit (Meridian Bioscience). Then, the extracted RNA was converted into cDNA using the commercial high‐capacity cDNA reverse transcription kit (Applied Biosystems, Thermo Fisher Scientific). Both RNA and cDNA concentrations were measured using a Nano‐Drop Spectrophotometer (Thermo Scientific NanoDrop 1000 Spectrophotometer).

RT‐qPCR assay was performed targeting the FCoV‐specific 7b gene using the primer described by Emmler et al.^13^ A total of 25 µL of PCR reaction mixture was prepared, which contained 12.5 µL of SYBR Green real‐time PCR master mix (Bio‐Rad), 1.0 µL of forward primer (5′‐GAT TTG ATT TGG CAA TGC TAG ATT T‐3′) and 1.0 µL of reverse primer (5′‐ACC AAT CAC TAG ATC CAG ACG TTA GCT‐3′), 2 µL of template cDNA, and the remaining 8.5 µL of nuclease‐free water to prepare the final reaction volume. A two‐step real‐time PCR was performed with an initial denaturation at 95°C for 5 min, followed by 40 cycles of 95°C for 5 s and 60°C for 1 min. Duplicate tests were performed for each sample, and RNase‐free water was used as a negative control in every reaction. Furthermore, the amplified PCR products were visualised on 2% agarose gel through a gel electrophoresis system using a 100 bp DNA marker (Figure S2). Furthermore, the viral identity was confirmed by sequencing the RT‐qPCR amplicons, which showed high similarity to reference FCoV sequences in GenBank. Basic local alignment search tool analysis revealed no cross‐reactivity with SARS‐CoV‐2 or other coronaviruses, confirming the specificity of the assay. After confirming FCoV infection, testing for additional viral co‐infections such as feline retroviruses (feline leukaemia virus and feline immunodeficiency virus) was not performed in the cats included in this study.

### Propagation of FCoV and observation of cytopathic effect

Vero cells (Vero CCL‐81, BSL) at passages 24–32 were used for virus propagation. Briefly, frozen stocks were rapidly thawed at room temperature, gently resuspended in complete growth medium containing foetal bovine serum (FBS), and seeded into T75 culture flasks (Sigma‒Aldrich) for recovery. Vero cells were cultured in Dulbecco's modified Eagle medium (DMEM) supplemented with 10% FBS, 1% antibacterial (100 U/mL penicillin and 100 µg/mL streptomycin) and 0.5% antifungal (250 µg/mL amphotericin B) components. The cultured cells were maintained in a humidified atmosphere of 5% CO_2_ at 37°C for up to 5 days until they reached confluence of approximately 90%.

**FIGURE 1:**
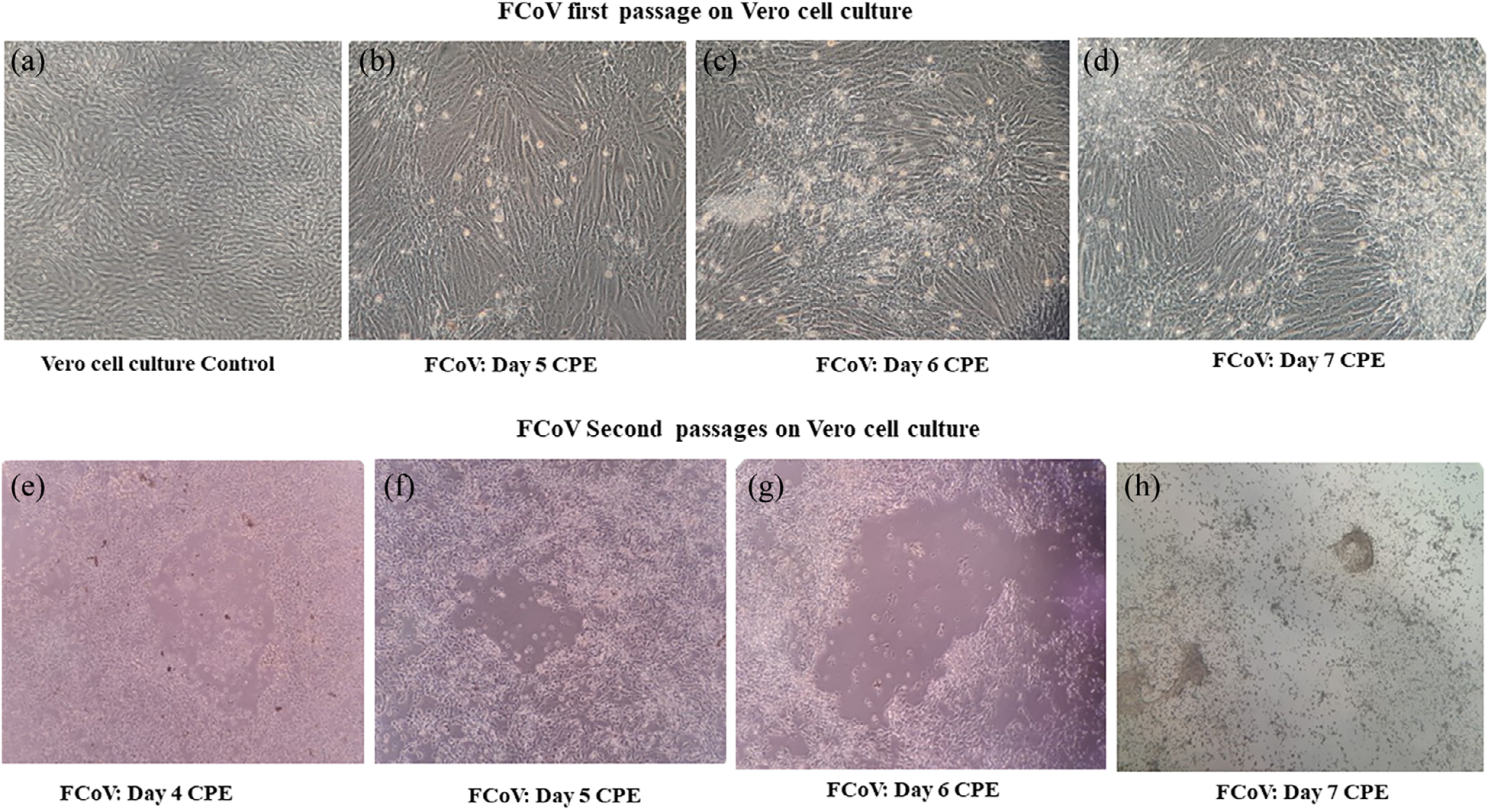
Cytopathic effects (CPEs) observed in cell culture. The CPE was observed in Vero cell monolayers following inoculation with peritoneal effusion samples. Panel (a) depicts the control Vero cell confluent monolayer that was not exposed to any sample or virus, representing a healthy, uninfected culture. First passage (b–d): initial CPE, characterised by rounding of a few cells, was observed on day 5 post‐infection (pi), with a gradual increase in cell rounding on days 6 and 7 pi. Second passage (e–h): following re‐inoculation with supernatant from the first passage, prominent CPE appeared as early as day 2 pi, with characteristic features including cell rounding, shrinkage, detachment, clearing and death of cells from the monolayer. FCoV, feline coronavirus.

Then, RT‐qPCR confirmed that the FCoV sample (500 µL peritoneal fluid) was inoculated (Figure 1a), and the cell culture flasks were incubated at 37°C in a 5% CO_2_ incubator. Prior to infection, the Vero cells were gently washed with phosphate‐buffered saline to remove any dead cells and residual medium. The cells were incubated with virus and 2 mL of DMEM at 37°C for 1 h to allow viral attachment to cells, with gentle swinging of the cell culture flask every 15 min to ensure uniform distribution of the virus. The infected Vero cell cultures were observed at every 24‐h interval post‐infection (pi) under an inverted microscope to document any evidence of cytopathic effect (CPE). On the 7th day, supernatant from the first passage was collected and inoculated into fresh Vero cell monolayers for the second passage. The progression of CPE was documented regularly till 7 days pi. Uninfected Vero cell cultures (approximately 90% confluence) were used as a negative control throughout the virus propagation. After that, CPE was assessed by observing morphological alteration, including cell rounding, shrinkage, detachment, monolayer clearing and cell death under an inverted microscope at 24‐h intervals for up to 7 days pi. Finally, RT‐qPCR was performed to confirm FCoV presence and replication in Vero cell cultures. Viral replication across passages was assessed by evaluating cell cultures at days 5 and 7 pi, with the corresponding cycle threshold (Ct) values for each passage from all three isolations incorporated in Table S5.

### FCoV amplicon sequencing and phylogenetic analysis

**FIGURE 2:**
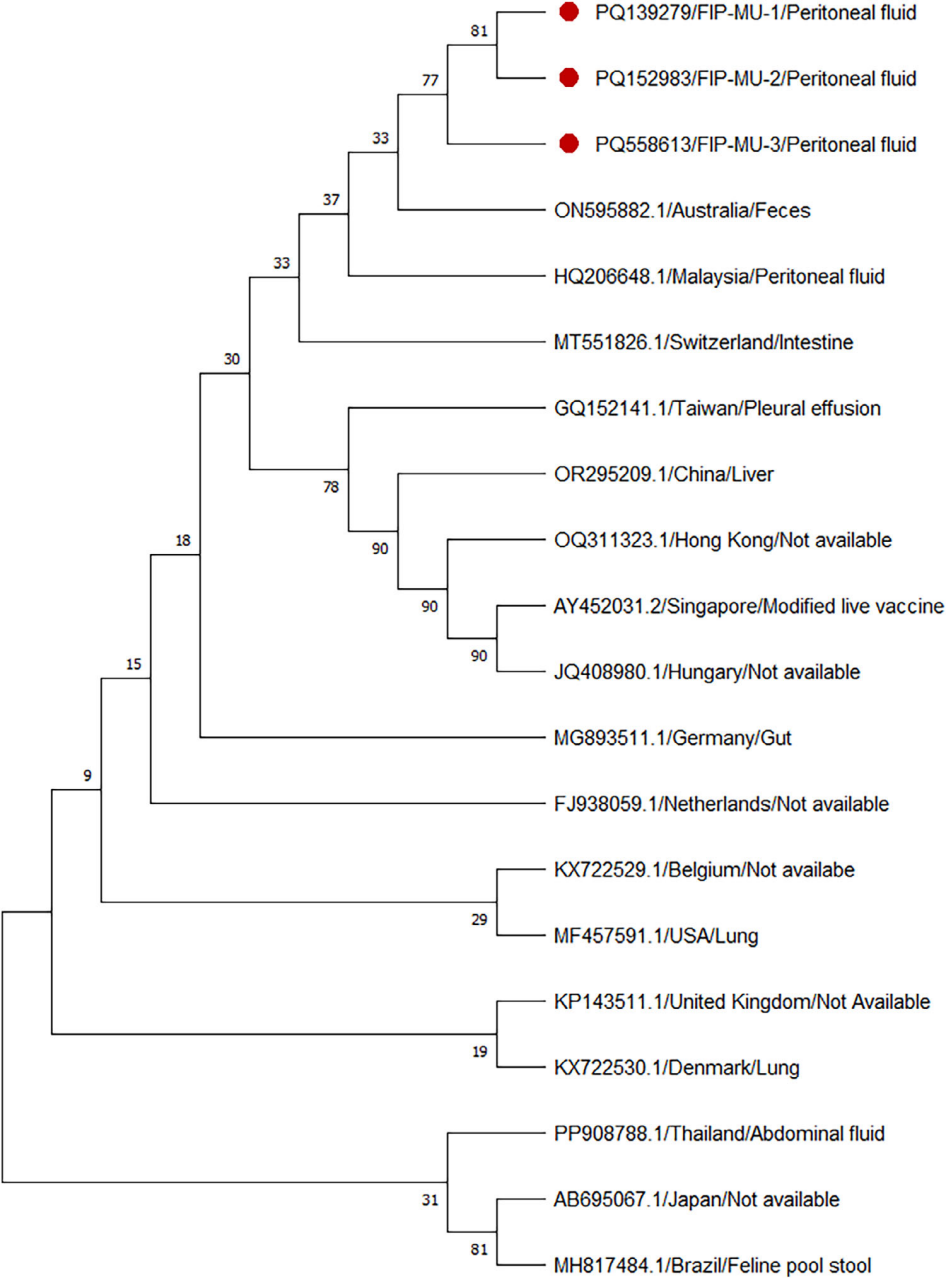
Phylogenetic analysis of feline coronavirus (FCoV). Phylogenetic analysis of FCoV sequences was performed using the 7b gene. The phylogenetic tree was constructed using the neighbour‐joining method in MEGA 11 with 1000 bootstrap replicates. Feline coronavirus sequences incorporated in the current analysis are identified by their GenBank accession number. Sequences marked with a solid red circle were obtained from cats with feline infectious peritonitis in the current study.

The amplified PCR products from the original effusion samples were purified using the PureLink Quick Gel Extraction Kit (Thermo Fisher Scientific) and submitted to the State Agricultural Biotechnology Centre, MU, for bidirectional Sanger sequencing of the target gene following standard protocols. The consensus sequence data were submitted to GenBank (www.ncbi.nlm.nih.gov/WebSub/) and the accession numbers (PQ139279, PQ152983 and PQ558613) were obtained. Furthermore, all three FCoV sequences of this study, along with 17 other sequences from different countries, were obtained from NCBI GenBank (Figure 2). All the sequences were aligned using the Multiple Sequence Comparison by Log‐Expectation algorithm in MEGA 11 version. The neighbour‐joining method was applied to construct the phylogenetic tree with a bootstrap value of 1000 replicates, and distances were calculated using the Kimura model.^14^ Following alignment, pairwise nucleotide identity was calculated to quantitatively assess the genetic similarity among the three sequences, providing insight into their genetic relatedness and potential divergence.

## RESULTS

### Clinical features of FIP‐affected cats

Three cases with clinical signs consistent with FIP were recruited (see Table S1). Among them, predominant clinical features were abdominal distension (ascites), respiratory distress with abnormal lung sounds (rales), icterus and cloudy cornea (Table S1).

### Haematology, serum biochemistry and IFAT of FCoV‐infected cats

Each FIP‐affected cat had supportive details in haematology and serum biochemistry results (see Table S2). The haematology profiles revealed mild neutrophilia and lymphopenia in cat 1, leukopenia, neutropenia with regenerative left shift, and lymphopenia in cat 2, and lymphopenia in cat 3. Only cat 2 has a mild, microcytic and normochromic anaemia. The blood serum biochemistry results (Table S2) showed a mild (cat 1) to moderate (cat 2) to severe (cat 3) increase in creatine kinase (CK) activity and a mild increase in aspartate aminotransferase (AST) activity consistent with myocyte damage. All patients had hyperbilirubinaemia, while hyperglycaemia was present in two out of three cats. Hyperproteinaemia and hypoalbuminaemia were common findings in all patients, while hyperglobulinaemia was observed in two of the three cats, reflecting systemic inflammation. Hypertriglyceridaemia was observed in two out of three cats, probably secondary increased in lipo‐mobilisation. The FCoV‐specific antibody titres were more than 1:2560 in all the three cats, as estimated by IFAT, and were considered seropositive. All the cats tested negative for *T. gondii* IgM antibodies by IFAT.

### Urinalysis of FIP cats

Urinalysis results are included in Table S4 for completeness. Notably, leakage of RBCs, leukocytes, epithelial cells and protein was detected in the urine of all three clinical cases. Moreover, marked bilirubinuria was observed in cats 2 and 3, while crystals in urine were detected in cats 1 and 3. All the samples were found to be free from bacterial and fungal growth (Table S3).

### Cytology and Rivalta test of peritoneal effusion

The peritoneal fluid was found to be yellowish and viscous in consistency in all three cases. Mixed inflammatory cells, such as neutrophils, lymphocytes and macrophages, were identified in the cytological examination (Table S4), with a predominance of non‐degenerate neutrophils (Figure S1a). Although neutrophils were only 28% in cat 3, it showed higher percentages of lymphocytes (12%) and macrophages (60%). Additionally, the peritoneal fluid smear showed a highly cellular, well‐preserved pale pink granular background containing rare erythrocytes (Figure S1b). All effusion fluids from FIP‐affected cats had a high protein content (Table S4). No overtly neoplastic cells or infectious agents such as bacteria and fungi were identified under the microscope, or in culture media. The Rivalta test was found positive in all peritoneal samples, indicating the presence of inflammatory exudate.

### FCoV propagation and observation of CPE

In Vero cell culture, CPEs characterised by mild cellular rounding were first evident on day 5 pi, with progressive intensification observed on days 6 and 7 pi (Figure 1b‒d). Upon second passage in Vero cells, using the RT‐qPCR confirmed FCoV‐positive supernatant from the first passage, characteristic CPE became visible by day 2 pi. The characteristic features of CPE, including cell rounding, shrinkage, detachment, clearing of the monolayer and cell death, were observed on days 5, 6 and 7 pi (Figure 1e‒h). Non‐inoculated control Vero cell culture‐maintained confluence and normal morphology up to day 10, with no CPE observed, and RT‐qPCR performed on supernatants at days 5 and 7 confirmed the absence of FCoV (Table S5).

### RT‐qPCR and phylogenetic findings

All three peritoneal effusion samples as well as the supernatants from passage 1 and passage 2 Vero cell cultures (days 5 and 7 pi) tested positive for FCoV by RT‐qPCR, with gel electrophoresis showing a distinct 102 bp band (Figure S2) and corresponding Ct values reported in Table S5. Phylogenetic analysis demonstrated that all three sequences were positioned within the same clade, indicating a shared evolutionary lineage (Figure 2). Moreover, these sequences showed close alignment with FCoV sequences retrieved from GenBank from different countries, including Australia, Malaysia, Switzerland and Taiwan, as supported by strong bootstrap values (Figure 2). Furthermore, pairwise nucleotide analysis of the three sequences revealed variable levels of similarity. Cats 1 and 2 were closely related, sharing 92.86% identity (78/84 nucleotides). In contrast, cat 3 was more divergent, exhibiting 63.10% and 64.29% identity with cats 1 and 2, respectively. These findings suggest that cat 3 may represent a distinct variant compared to the other two sequences.

## DISCUSSION

In this study, we performed a systematic set of laboratory tests to achieve a consistent and confirmatory diagnosis of FIP and carried out in vitro propagation of FCoV in Vero cells to evaluate its susceptibility and adaptation to a non‐feline cell line. This approach not only confirmed the presence of FCoV in clinically affected cats but also evaluated the virus's ability to replicate in an alternative non‐feline cell line. Feline coronavirus infection in cats is associated with various nonspecific clinical conditions and clinicopathologic abnormalities, making the clinical diagnosis of FIP challenging, particularly when based solely on clinical examination. The FCoV‐affected cats exhibited abdominal distension, abnormal lung sounds, icterus and cloudy corneas, which are among the most frequently observed clinical signs of FIP.^1^, ^8^, ^15^ Recognition of the frequent and common clinical features of FIP provides essential initial guidance for clinicians in selecting appropriate samples, diagnostic tests and urgent supportive treatments. In this study, the clinical presentation of the three cats served as a critical first step for case selection, reporting the value of clinical assessment in resource‐limited settings where advanced diagnostic facilities are often unavailable.

The haematological profile of FIP‐affected cats typically shows marked neutrophilia, which is associated with an inflammatory response driven by systemic viral infection.^9^ All cats in the study exhibited a marked reduction in lymphocyte counts. This feature is consistent with previous reports describing lymphopaenia and lymphoid depletion in the spleen and lymph nodes of FIP‐infected cats.^16^ Cats infected with FCoV typically exhibit follicular hyperplasia in peripheral lymph nodes, highlighting the immune dysregulation associated with the infection.^16^

The serum biochemistry profile indicated significant systemic involvement in clinical disease. Elevated CK and AST levels are strongly associated with muscle and liver damage in the infected host, likely resulting from the widespread inflammatory response and tissue damage caused by viral replication, which is characteristic of FIP.^17^ Hyperbilirubinaemia in FIP‐affected cats could be attributed to liver dysfunction, which is commonly observed in chronic inflammatory conditions.^17^ Moreover, the acute‐phase protein such as serum amyloid‐A is significantly raised during systemic inflammation and is considered a useful biomarker for differentiating FIP from other clinically similar disease conditions.^18^ Seropositive results with high antibody titres are often associated with viral shedding, and suggest active or chronic infection with FCoV.^19^ Moreover, the detection of RBCs, leukocytes and protein in the urine indicates glomerular damage as well as urinary tract inflammation, which could be associated with complications of FCoV infection.^19^ Marked bilirubinuria is commonly observed in FIP‐affected cats, reflecting hepatobiliary damage, as previously reported by Hugo and Heading.^20^ However, reference values for laboratory test results may vary depending on the diagnostic guidelines and methodologies recommended by the manufacturer, as well as animal‐specific factors such as breed, age, sex and genetic background. These inherent variations should be considered when interpreting the clinicopathological findings of the three cats in this study and represent a study limitation.

Cytological examination of effusion fluid showed inflammatory cells as well as intact and lysed erythrocytes, indicating immune‐mediated vasculitis, a hallmark of FIP.^15^ Moreover, the high protein and bilirubin content represent increased vascular permeability and haemolysis.^21^ These clinicopathological features, commonly observed in FIP cases due to immune complex deposition in the liver and kidneys, cause organ dysfunction.^22^ Although IFAT provides supportive serological evidence for FIP, it cannot differentiate between FECV and the pathogenic FIPV. Nevertheless, when used alongside haematology, biochemistry, RT‐qPCR and sequencing, IFAT provides valuable supportive information that strengthens the overall diagnostic assessment.

The characteristic features of CPE observed uniformly in the cell culture flasks, including cell rounding, shrinkage and detachment are consistent with the typical signs of coronavirus infection in permissive cell lines, as described by Ruggieri et al.^23^ In the second passage, the early appearance of CPE (day 2 pi) provided strong evidence of a higher viral copy number carried over from the first passage, as well as improved viral adaptation and permissiveness in the Vero cell line. This observation, together with the consistent decline in Ct values across passages, confirms that FCoV actively replicated in Vero cells rather than persisting as residual viral nucleic acid. Although FCoV‐2 can replicate in feline‐derived cell lines such as fcwf‐4, CRFK or FCWF,^23^, ^24^, ^25^ the limited accessibility of these cell lines restricts their widespread use. Typically, FCoV exhibits limited in vitro replication in all types of cell lines.^24^ Since the FCoV isolate successfully adapted to and propagated in the Vero cell line, it is likely indicative of genotype 2, which is well known for its broader cell line susceptibility.^24^ Efficient in vitro propagation of FCoV in non‐feline cell lines, such as Madine–Darby Canine Kidney and Vero cells, has also been reported in previous studies.^24^, ^25^, ^26^ In the current study, FCoV was successfully propagated in Vero cell culture, which exhibited distinct cytopathic changes. While the exact mechanisms underlying Vero cell susceptibility to FCoV remain unclear, one possible explanation is receptor‐mediated entry and the broad permissiveness of Vero cells to various coronaviruses. Feline coronavirus is known to utilise aminopeptidase N (APN/CD13) as its cellular receptor, which is also expressed in primate‐derived Vero cells, thereby facilitating viral binding and entry.^27^ In addition, Vero cells are deficient in type I interferon production, reducing their innate antiviral defense and enhancing viral replication.^28^ In contrast, the restricted propagation of FCoV‐1 in non‐feline cell lines is likely attributable to its reliance on an as‐yet unidentified cellular receptor.^29^ The spike protein–receptor interaction is a key determinant of coronavirus cell tropism.^29^ To infect a new host cell or an in vitro cell culture, coronaviruses must adapt to the cell surface receptor, either through mutation or recombination with a related coronavirus.^30^ FCoV‐1 may fail to replicate in Vero cells due to an inability to adapt to available cell‐surface receptors, whereas genotype 2 is potentially permissive, likely because Vero cells express APN/CD13, a known receptor for genotype 2.^27^, ^30^ This difference highlights the importance of receptor compatibility in determining host cell susceptibility and the successful in vitro propagation of FCoV. Therefore, FCoV‐1 remains less well understood, partly because of difficulties in propagating it in cell culture. Nevertheless, FCoV‐1 has only been propagated infrequently, with growth reported in long‐term feline intestinal epithelial cultures derived from ileocytes and colonocytes.^31^ Although both genotypes of FIPV are often assumed to behave similarly throughout most of the infection process, it remains unclear whether the same or different mutations are responsible for the biotype switch.^30^ Therefore, further studies on FCoV‐1 are urgently needed, as such research would be crucial for understanding the mechanisms underlying FCoV pathogenesis and the development of progressive disease. However, current in vitro findings provide valuable insights into the potential utility of Vero cells as an alternative culture system for FCoV propagation, particularly in resource‐limited settings where feline‐specific cell lines may not be readily available. Moreover, the successful adaptation and propagation of FCoV in non‐feline cells may support a range of broader applications, including generating sufficient viral RNA for complete genome sequencing to obtain in‐depth genetic insights, studying host–virus interactions, characterising antiviral resistance, and establishing a practical platform for antiviral drug screening. In the context of vaccine development, the greatest promise of this study lies in generating naturally circulating (wild‐type) viral material that can serve as a foundation for antigen production, including the formulation of inactivated or attenuated vaccines. Although Vero cells are not the natural host for FCoV, successful isolation in this cell line provides a valuable platform for preliminary investigations, particularly in resource‐limited laboratories. Moreover, Vero cells have been widely used in virology due to their high permissiveness to a variety of viruses and their ability to produce clear CPEs, which facilitates the study of viral replication dynamics. Establishing FCoV in Vero cells enables controlled in vitro experiments to inform future research, and Vero‐based propagation serves as a practical model that complements host‐specific studies and supports further investigations. The current adaptation indicates the potential for FCoV to exhibit cross‐species tropism and enhanced permissiveness in diverse cell lines. Despite their value for viral propagation, Vero cells may be unsuitable for pathogenesis studies due to the absence of feline‐specific immune receptors and the impaired production of type I interferons, which limits meaningful insights into host immune responses. Since Shah et al. reported propagation of a field strain of FIPV without detailing the CPE and the replication kinetics, this study provides new insights into these virological characteristics.^26^ To our knowledge, this is the unprecedented documented report demonstrating the successful propagation and characteristic CPE of FCoV in Vero cell culture. Although FCoV successfully replicated and exhibited characteristic CPE suggestive of FCoV‐2, detailed serological tests, such as virus neutralisation tests, are required in future studies. While multiplex PCR was not performed to rule out other viral or protozoal co‐infections in the cats, RT‐qPCR of every cell culture flask at days 5 and 7 pi showed continuously decreasing Ct values, providing strong evidence of FCoV replication. In future studies, we plan to perform metagenomic analysis or multiplex PCR to detect any potential co‐infections. In addition to the Vero cell line, large‐scale in vitro studies using a variety of non‐feline cell lines are highly recommended to better understand the cell tropism of FCoV.

Furthermore, phylogenetic analysis showed that the FCoV partial sequences cluster within the same clade, suggesting a shared evolutionary origin and minimal genetic divergence. The close alignment of these sequences with isolates from diverse countries, including Malaysia, Switzerland, Taiwan and the southeast coast of Australia highlights their genetic relatedness.^1^ Although the short sequence fragment used in this study is insufficient for robust phylogenetic analysis or comprehensive virus typing, future studies should prioritise complete genome sequencing to enable precise phylogenetic analysis and definitive genotype determination. Moreover, it is essential for understanding the detailed genomic features of FCoV, including its genetic diversity, transmission dynamics and evolutionary patterns. The observed variation in pairwise nucleotide identity among the three sequences provides insight into their evolutionary relationships. The high similarity between cats 1 and 2 (92.86%) suggests that these sequences likely originate from closely related viral strains or share a recent common ancestor, reflecting limited genetic divergence. In contrast, the considerably lower identity of cat 3 with cats 1 and 2 (63.10% and 64.29%, respectively) indicates that cat 3 represents a genetically distinct variant, suggesting the circulation of multiple viral strains within the natural environment. Such genetic heterogeneity has important implications for viral transmission dynamics, and diagnostic detection, as divergent variants may differ in virulence or antigenicity.^32^ Therefore, continuous molecular surveillance is essential to elucidate the diversity of circulating wild strains, which is critical for effective epidemiological monitoring and the development of targeted control strategies.

## CONCLUSION

This study confirms the presence of FCoV infection using multiple laboratory findings consistent with FIP in cats. The combination of a positive Rivalta test, high protein and bilirubin content in effusion fluid, and the absence of bacterial or fungal growth may be recommended as an early diagnostic approach for FIP, which can assist in immediate treatment decisions. The successful adaptation and propagation of FCoV in Vero CCL‐81 cells, with characteristic CPE, indicate a reliable, and promising approach for isolating wild strains responsible for FIP. This model could facilitate future research on FCoV genomics, targeted antiviral screening and the in vitro development of effective vaccines using non‐feline cell lines. Additionally, this methodological approach, particularly the use of Vero cell culture for FCoV propagation, may have broader applicability, as similar mechanisms of adaptation and propagation could potentially be applied to other coronaviruses or related viruses affecting diverse host species.

## AUTHOR CONTRIBUTIONS

Eaftekhar Ahmed Rana: Conceptualization; Methodology; project administration, investigation, validation; data curation; formal analysis, visualization, software, writing‐original draft, writing review and editing. Mahfuza Akther: Methodology and project administration. Susan Beetson: Sample collection; resources; investigation. Subir Sarker: Validation; writing review and editing. Gabriele Rossi: Sample collection; resources; investigation; validation; writing review and editing. Jasim Muhammad Uddin: Conceptualization; investigation; resources; validation; writing review and editing and supervision.

## CONFLICTS OF INTEREST

The authors declare they have no conflicts of interest.

## FUNDING INFORMATION

The authors declare they have no conflicts of interest.

## ETHICS STATEMENT

The study was conducted in accordance with the ethical guidelines of the Animal Ethics Committee at Murdoch University for the diagnosis and investigation of animals at the Murdoch University Animal Hospital. The clinical cases were derived from the Murdoch University Animal Hospital, where the cats were presented for clinical diagnosis and treatment. Therefore, no specific approval number was applicable to this study.

## Supporting information



Supporting Information
**FIGURE S1**. Cytology smear of the effusion fluid illustrates a mixed inflammatory cell population, predominantly non‐degenerate neutrophils with a smaller proportion of macrophages (a). The highly cellular and well‐preserved pale pink granular background contains rare erythrocytes (b). No bacteria or fungi were observed on the smear. There is no evidence of cytophagia, erythrophagia or haemosiderin within macrophages. No overtly neoplastic cells were identified. The black arrow indicates the corresponding cytological lesions.

Supporting Information
**FIGURE S2**. Gel electrophoresis of amplified polymerase chain reaction (PCR) products of feline coronavirus (FCoV) isolates. The image shows specific amplification of FCoV products at 102 bp on a 2% agarose gel. Lane M represents the 100 bp DNA marker, while lanes L1–L2 correspond to the cat 1 sample, and lanes L3–L4 and L5–L6 correspond to the FCoV‐positive isolates from cats 2 and 3, respectively.

Supporting Information

Supporting Information

Supporting Information

Supporting Information

Supporting Information

## Data Availability

The authors confirm that the laboratory results supporting the findings of this study are provided within the article.
