# Supplementary figures and images for: Successful in vitro propagation of feline coronavirus from clinically diagnosed feline infectious peritonitis cases using Vero cells: A potential model for future research
Source: Vet Rec Open. 2026 Feb 25;13(1):e70030. doi: 10.1002/vro2.70030 (PMC12935566; doi:10.1002/vro2.70030)

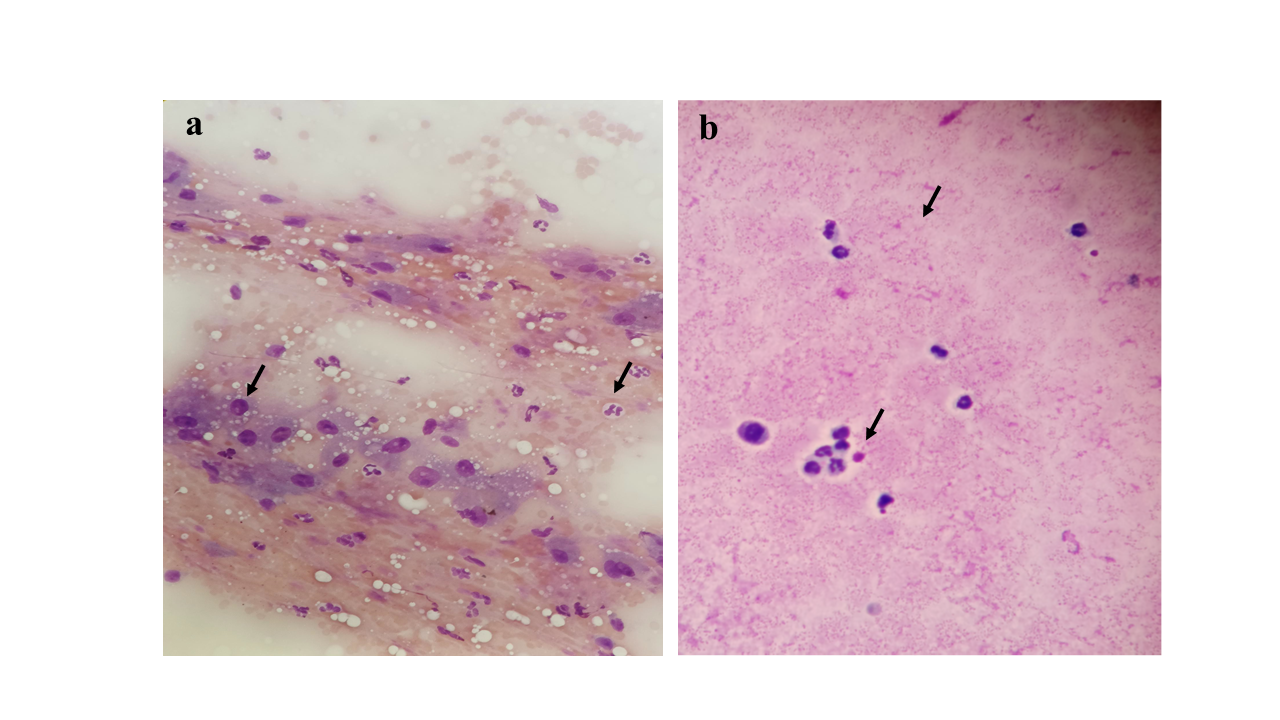

Supplement: Supplementary file 1 — Supporting Information FIGURE S1. Cytology smear of the effusion fluid illustrates a mixed inflammatory cell population, predominantly non‐degenerate neutrophils with a smaller proportion of macrophages (a). The highly cellular and well‐preserved pale pink granular background contains rare erythrocytes (b). No bacteria or fungi were observed on the smear. There is no evidence of cytophagia, erythrophagia or haemosiderin within macrophages. No overtly neoplastic cells were identified. The black arrow indicates the corresponding cytological lesions. [file VRO2-13-e70030-s006.png]

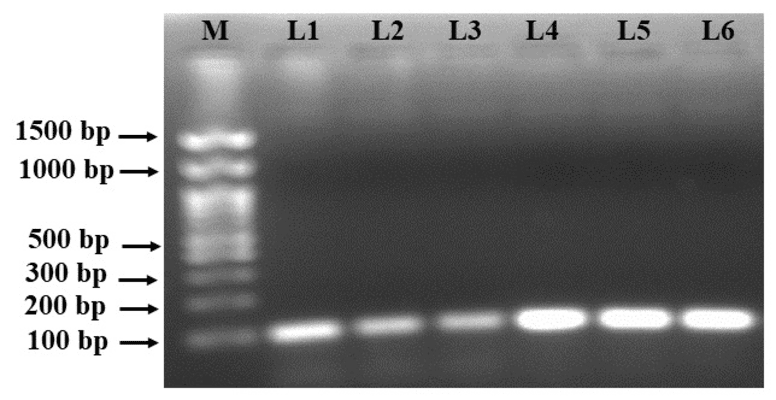

Supplement: Supplementary file 2 — Supporting Information FIGURE S2. Gel electrophoresis of amplified polymerase chain reaction (PCR) products of feline coronavirus (FCoV) isolates. The image shows specific amplification of FCoV products at 102 bp on a 2% agarose gel. Lane M represents the 100 bp DNA marker, while lanes L1–L2 correspond to the cat 1 sample, and lanes L3–L4 and L5–L6 correspond to the FCoV‐positive isolates from cats 2 and 3, respectively. [file VRO2-13-e70030-s003.png]
