# Supplementary material for: Successful in vitro propagation of feline coronavirus from clinically diagnosed feline infectious peritonitis cases using Vero cells: A potential model for future research
Source: Vet Rec Open. 2026 Feb 25;13(1):e70030. doi: 10.1002/vro2.70030 (PMC12935566; doi:10.1002/vro2.70030)
Supplement: Supplementary file 3 — Supporting Information [file VRO2-13-e70030-s002.docx]

**Supplementary Table 1: Clinical signs and symptoms observed at admission during the examination of cats affected by FIP.**

| **Sl no** | **Category** | **Physical examination findings** | **Clinical condition** | | |
| --- | --- | --- | --- | --- | --- |
|  |  |  | **^#^Cat-1** | **^#^Cat-2** | **^#^Cat-3** |
| 1 | Non-specific | Fever | Present (105.2°F) | Absent (102.6°F) | Present (104.3°F) |
| 2 |  | Anorexia | Present | Present | Present |
| 3 |  | Jaundice (icterus) | Present | Present | Present |
| 4 |  | Lethargy | Present | Present | Present |
| 5 |  | Weight loss/failure to gain weight | Absent | Present | Present |
| 6 |  | Pale mucous membranes | Absent | Present | Present |
| 7 |  | Lymphadenomegaly | Absent | Absent | Absent |
| 8 | Abdominal | Distension | Present | Present | Present |
| 9 |  | Ascites | Present | Present | Present |
| 10 |  | Diarrhoea | Absent | Absent | Absent |
| 11 |  | Lymphadenomegaly | ^*^Unknown | Absent | Absent |
| 12 | Respiratory | Respiratory distress, tachypnea | Present | Absent | Absent |
| 13 |  | Abnormal lung sound (rales) | Present | Absent | Present |
| 14 |  | Pleural effusion | Present | Absent | Absent |
| 15 | Cardiac | Pericardial effusion | ^*^Unknown | Absent | Absent |
| 16 | Reproductive | Scrotal effusion | Absent | Absent | Absent |
| 17 |  | Priapism | Absent | Absent | Absent |
| 18 | Neurological | Seizures | Absent | Absent | Absent |
| 19 |  | Abnormal mentation | Absent | Absent | Absent |
| 20 |  | Central vestibular signs (nystagmus, head tilt, circling, obtunded appearance and postural reaction deficits) | Absent | Present (obtunded) | Absent |
| 21 |  | Anisocoria | Absent | Absent | Absent |
| 22 |  | Ataxia | Absent | Absent | Absent |
| 23 |  | Tetra- or para-paresis | Absent | Absent | Absent |
| 24 |  | Incoordination | Absent | Absent | Absent |
| 25 | Ocular | Uveitis | Absent | Absent | Absent |
| 26 |  | Blindness | Absent | Absent | Absent |
| 27 |  | Hyphema | Absent | Absent | Absent |
| 28 |  | Corneal opacity | Present | Absent | Present |
| 29 |  | Retinal vasculitis/Retinal detachment | Retinitis | Absent | Absent |

**^#^**The first clinical case (designated as Cat-1) was a 12-month-old male Mixed Medium hair cat, the second case (designated as Cat-2) was a six-month-old male British Shorthair cat, and the third case (designated as Cat-3) was a 12-month-old male Domestic Shorthair cat.

*Unknown: During the clinical examination, the clinician did not confirm the presence of lymphadenomegaly and pericardial effusion in the FIP cats.
