# Supplementary material for: Successful in vitro propagation of feline coronavirus from clinically diagnosed feline infectious peritonitis cases using Vero cells: A potential model for future research
Source: Vet Rec Open. 2026 Feb 25;13(1):e70030. doi: 10.1002/vro2.70030 (PMC12935566; doi:10.1002/vro2.70030)
Supplement: Supplementary file 4 — Supporting Information [file VRO2-13-e70030-s005.docx]

**Supplementary Table 2: Haematology and serum biochemistry profiles of FIP affected cats.**

| **Sl no** | **Analysis** | **Results** | | | **Reference Interval** |
| --- | --- | --- | --- | --- | --- |
|  |  | **^#^Cat-1** | **^#^Cat-2** | **^#^Cat-3** |  |
| **Haematology profiles (complete blood count)** | |  |  |  |  |
| 1 | Total WBCs (x10^9/L) | 15.8 | 2.0 **↓** | 10.4 | 5.5 - 19.5 |
| 2 | Abs Bands (x10^9/L) | 0.2 | 0.48 **↑** | 0.21 | 0.0 - 0.3 |
| 3 | Abs Neutrophils (x10^9/L) | 14.5 **↑** | 1.5 **↓** | 9.8 | 2.5 - 12.5 |
| 4 | Abs Lymphocytes (x10^9/L) | 0.9 **↓** | 0.0 **↓** | 0.3**↓** | 1.5 - 7.0 |
| 5 | Abs Monocytes (x10^9/L) | 0.3 | 0.0 | 0.1 | 0.0 - 0.9 |
| 6 | Abs Eosinophils (x10^9/L) | 0.4 | 0.0 | 0.0 | 0.0 – 1.5 |
| 7 | Abs Basophils (x10^9/L) | 0.0 | 0.0 | 0.0 | 0.0 – 0.3 |
| 8 | Rel Bands (%) | 1 | 24 | 2 | - |
| 9 | Rel Neutrophils (%) | 92 | 73 | 94 | - |
| 10 | Rel Lymphocytes (%) | 6 | 1 | 3 | - |
| 11 | Rel Monocytes (%) | 2 | 2 | 1 | - |
| 12 | Rel Eosinophils (%) | 4 | 2 | 0 | - |
| 13 | Rel Basophils (%) | 0.0 | 1 | 0 | - |
| 14 | RBC (x10^12/L) | 6.95 | 5.24 | 7.25 | 5.00 – 10.00 |
| 15 | HGB (g/L) | 96.0 | 53.0 **↓** | 84.0 | 80.0 – 150.0 |
| 16 | HCT (L/L) | 0.28 | 0.16 **↓** | 0.28 | 0.24 – 0.45 |
| 17 | MCV (Fl) | 40 | 31 **↓** | 39 | 39 – 55 |
| 18 | MCH (pg) | 14 | 10 **↓** | 12**↓** | 13 – 17 |
| 19 | MCHC (g/L) | 343 | 331 | 300 | 300 – 360 |
| 20 | PLT Quant | Appear Adequate | Appear Adequate | Appear Adequate | - |
| 21 | PLT Morph | Appear normal | Enlarged | Appear normal | - |
| 22 | PLT Morph Numbers | 1+ | 2+ | 1+ | - |
| 23 | Thrombo Estimate (x10^9/L) | - | 140 | - | - |
| 24 | Nucleated RBCs (/100 WBCs) | 0 | 0 | 0 | - |
| **Biochemistry profiles** | |  |  |  |  |
| 1 | Creatine Kinase (U/L) | 1243.00 **↑** | 5800.00 **↑** | 12904.00**↑** | 50.00 - 200.00 |
| 2 | Aspartate Aminotransferase (AST) (U/L) | 96.00 **↑** | 196.00 **↑** | 392.00**↑** | 26.00 - 43.00 |
| 3 | Alanine Aminotransferase (ALT) (U/L) | 24.00 | 53.00 | 154.00↑ | 6.00 - 83.00 |
| 4 | Alkaline Phosphatase (ALP) (U/L) | 16.00 | 10.00 | 16.00 | 25.00 - 93.00 |
| 5 | Gamma GT (U/L) | < 1.00 | 1.20 | <1.00 | 1.00 - 5.00 |
| 6 | Bilirubin (umol/L) | 45.00 **↑** | 81.00 **↑** | 35.00**↑** | 2.00 - 17.00 |
| 7 | Urea (mmol/L) | 6.20 **↓** | 4.20 **↓** | 5.30**↓** | 7.00 - 10.70 |
| 8 | Creatinine (umol/L) | 64.00 **↓** | 18.00 **↓** | 54.00**↓** | 70.00 - 159.00 |
| 9 | Sodium (mmol/L) | 147.00 | 140.80 **↓** | 143.80**↓** | 145.00 - 156.00 |
| 10 | Potassium (mmol/L) | 3.93 | 2.34 **↓** | 4.00 | 3.80 - 5.20 |
| 11 | Sodium/Potassium Ratio | 43.37 | 60.17 | 35.95 |  |
| 12 | Chloride (mmol/L) | 114.00 | 103.20 **↓** | 113.40 | 110.00 - 123.00 |
| 13 | Glucose (mmol/L) | 8.5 **↑** | 13.2 **↑** | 3.9 | 3.8 - 6.1 |
| 14 | Lipase DGGR (U/L) | 9.00 | 15.00 | 10.00 | 0.00 - 26.00 |
| 15 | Total protein (g/L) | 71.00 | 49.00 **↓** | 90.00**↑** | 54.00 - 78.00 |
| 16 | Albumin (g/L) | 19.00**↓** | 16.40 **↓** | 17.80**↓** | 21.00 - 39.00 |
| 17 | Globulin (g/L) | 54**↑** | 33 | 72**↑** | 25 - 50 |
| 18 | A/G Ratio | 0.35**↓** | 0.50 | 0.25**↓** | 0.50 - 1.20 |
| 19 | Calcium (mmol/L) | 2.14 | 1.89 | 1.99 | 1.50 - 2.60 |
| 20 | Phosphate (mmol/L) | 2.38 | 1.28 **↓** | 2.18 | 1.40 - 2.50 |
| 21 | Cholesterol (mmol/L) | 3.67 | 3.50 | 4.00**↑** | 2.50 - 3.90 |
| 22 | Triglycerides (mmol/L) | 1.89 **↑** | 2.81 **↑** | 0.79 | 0.60 - 1.20 |
| 23 | Serum Amyloid A (mg/L) | 42.70**↑** | 38.90 **↑** | 50.00**↑** | <=6.00 |

**Note:** RBC: Red blood cell; HGB: Hemoglobin; HCT: Hematocrit; MCV: Mean corpuscular volume; MCHC: Mean corpuscular hemoglobin concentration; PLT Quant: Platelet counts; PLT Morph: Platelet size and morphology. ↑: Increase; ↓: Decrease. Cat-1: Mixed Mediumhair cat, Cat-2: British Shorthair cat, Cat-3: Domestic Shorthair cat.

#The first clinical case (designated as Cat-1) was a 12-month-old male Mixed Medium hair cat, the second case (designated as Cat-2) was a six-month-old male British Shorthair cat, and the third case (designated as Cat-3) was a 12-month-old male Domestic Shorthair cat.
