# Supplementary material for: Successful in vitro propagation of feline coronavirus from clinically diagnosed feline infectious peritonitis cases using Vero cells: A potential model for future research
Source: Vet Rec Open. 2026 Feb 25;13(1):e70030. doi: 10.1002/vro2.70030 (PMC12935566; doi:10.1002/vro2.70030)
Supplement: Supplementary file 5 — Supporting Information [file VRO2-13-e70030-s001.docx]

**Supplementary Table 3: Analysis of urine samples from cats affected by FIP.**

| **Sl no** | **Analysis** | **Results** | | |
| --- | --- | --- | --- | --- |
|  |  | **^#^Cat-1** | **^#^Cat-2** | **^#^Cat-3** |
| 1 | Specific Gravity (SG) | 1.046 | 1.014 | 1.050 |
| 2 | pH | 8.50 | 8.50 | 6.00 |
| 3 | Protein | 1+ | Trace | 3+ |
| 4 | Blood | Negative | Negative | Trace-intact |
| 5 | Ketone | Negative | Negative | Negative |
| 6 | Bilirubin | Negative | 2+ | 3+ |
| 7 | Glucose | Negative | Negative | Trace |
| 8 | Leucocytes (/uL) | 18 | 18 | 10 |
| 9 | RBC's (/uL) | 1 | 9 | 7 |
| 10 | Epithelial cells (/uL) | 27 | 9 | 47 |
| 11 | Crystals | Struvite | None Observed | Needle Shaped |
| 12 | Casts | None Observed | None Observed | None Observed |
| 13 | Debris | Occasional | Occasional | 1+ |
| 14 | Bacteria | None Observed | None Observed | None Observed |
| 15 | Fungal Elements | None Observed | None Observed | None Observed |

**^#^**The first clinical case (designated as Cat-1) was a 12-month-old male Mixed Medium hair cat, the second case (designated as Cat-2) was a six-month-old male British Shorthair cat, and the third case (designated as Cat-3) was a 12-month-old male Domestic Shorthair cat.
