# Supplementary material for: Successful in vitro propagation of feline coronavirus from clinically diagnosed feline infectious peritonitis cases using Vero cells: A potential model for future research
Source: Vet Rec Open. 2026 Feb 25;13(1):e70030. doi: 10.1002/vro2.70030 (PMC12935566; doi:10.1002/vro2.70030)
Supplement: Supplementary file 6 — Supporting Information [file VRO2-13-e70030-s007.docx]

**Supplementary Table 4: Cytology and microbiological assessment of effusion fluid of FIP affected cats.**

| **Sl. No** | **Analysis (Direct smear and cytospin are examined)** | **Results** | | |
| --- | --- | --- | --- | --- |
|  |  | **^#^Cat-1** | **^#^Cat-2** | **^#^Cat-3** |
| 1 | Consistency | Viscous | Viscous | Viscous |
| 2 | Colour | Light yellow to orange | Light yellow to orange | Light yellow to orange |
| 3 | Cell count | 17,850/μL | 1.5 x 10^9/L | 1.1 x 10^9/L |
| 4 | RBC | 1,350/μL | Lysed | Lysed and nucleated |
| 5 | Neutrophils | 93% | 85% | 28% |
| 6 | Lymphocytes | Not detected | 1% | 12% |
| 7 | Macrophages | 7% | 14% | 60% |
| 8 | Eosinophils | 0% | 0% | 0% |
| 9 | Specific Gravity (SG) | 1.023 | 1.027 | 1.038 |
| 10 | Protein | 64 g/L | 51 g/L | 74 g/l |
| 11 | Fluid bilirubin | 22 umol/L | 58 umol/L | 18 umol/L |
| 12 | Rivalta test | Positive | Positive | Positive |
| 13 | Gram staining | No bacteria seen | No bacteria seen | No bacteria seen |
| 14 | Culture on 5% Sheep blood agar (Aerobic culture) | No growth | No growth | No growth |
| 15 | Fungal culture (Sabouraud dextrose agar) | No growth | No growth | No growth |
| 16 | Neoplastic cells | Not observed | Not observed | Not observed |

**^#^**The first clinical case (designated as Cat-1) was a 12-month-old male Mixed Medium hair cat, the second case (designated as Cat-2) was a six-month-old male British Shorthair cat, and the third case (designated as Cat-3) was a 12-month-old male Domestic Shorthair cat.
