# Supplementary material for: Successful in vitro propagation of feline coronavirus from clinically diagnosed feline infectious peritonitis cases using Vero cells: A potential model for future research
Source: Vet Rec Open. 2026 Feb 25;13(1):e70030. doi: 10.1002/vro2.70030 (PMC12935566; doi:10.1002/vro2.70030)
Supplement: Supplementary file 7 — Supporting Information [file VRO2-13-e70030-s004.docx]

**Supplementary Table 5: RT-qPCR** **cycle threshold (Ct) values demonstrating viral replication dynamics across sequential passages of Vero cell cultures from three independent FCoV isolations.**

| **Peritoneal effusion/Virus infection on cell culture** | **^#^Cat no** | **RT-qPCR results** | **Initial Ct value** | **Ct value of cell culture derived samples** | | **RT-qPCR results of control sample** |
| --- | --- | --- | --- | --- | --- | --- |
|  |  |  |  | **Day 5 ^*^pi** | **Day 7 ^*^pi** |  |
| Peritoneal effusion | Cat-1 | Positive | 17.80 | - | - | Negative |
|  | Cat-2 | Positive | 19.30 | - | - | Negative |
|  | Cat-3 | Positive | 22.50 | - | - | Negative |
| First passage | Cat-1 | Positive | 17.80 | 12.90 | 11.60 | Negative |
|  | Cat-2 | Positive | 19.30 | 16.50 | 14.20 | Negative |
|  | Cat-3 | Positive | 22.50 | 18.0 | 15.30 | Negative |
|  | Control Vero cell culture | ^*^Negative | - | 38.90 | 38.80 | Negative |
| Second Passage | Cat-1 | Positive | 11.60 | 6.20 | 4.70 | Negative |
|  | Cat-2 | Positive | 14.20 | 9.10 | 5.80 | Negative |
|  | Cat-3 | Positive | 15.30 | 8.0 | 5.90 | Negative |
|  | Control cell culture | ^*^Negative | - | 39.0 | 38.80 | Negative |

#The first clinical case (designated as Cat-1) was a 12-month-old male Mixed Medium hair cat, the second case (designated as Cat-2) was a six-month-old male British Shorthair cat, and the third case (designated as Cat-3) was a 12-month-old male Domestic Shorthair cat. Positive: Any sample with a Ct value ≤37 was considered positive for the presence of FCoV; Negative: Any sample with a Ct value ≥38 was considered negative, indicating absence of FCoV; ^*^Pi: post infection. ^*^Negative: Used as an uninfected control cell culture and not exposed to any samples or virus.
